# Supplementary material for: The oldest three-dimensionally preserved vertebrate neurocranium
Source: Nature. 2023 Sep 20;621(7980):782–7. doi: 10.1038/s41586-023-06538-y (PMC10533405; doi:10.1038/s41586-023-06538-y)
Supplement: Supplementary file 1 — Comments on histology, phylogenetic analysis notes and references. [file 41586_2023_6538_MOESM1_ESM.pdf]

nature portfolio

https://doi.org/10.1038/s41586-023-06538-y

Supplementary information

The oldest three-dimensionally preserved vertebrate neurocranium

In the format provided by the authors and unedited

1.Comments on histology

Histology.
The histology of the dermal armour of *Eriptychius* has been described on a number of occasions, most extensively by Denison[5], Ørvig[37,56,] and Smith and Hall[57]. Although the terminology varies between these authors, the tesserae are comprised of acellular bone (aspidin), surmounted by ornamented tubercles formed from extremely coarse tubular dentine. Hypermineralised superficial caps to the tubercles are almost wholly absent and have only been convincingly illustrated by Smith and Hall[57] (fig 8E-F), this was interpreted as enamel due to the sharp boundary with the underlining dentine. The dermal elements in the articulated specimen of *Eriptychius* have a histological structure matching this in our scan data (Fig. S3).

Globular calcified cartilage is reasonably common in thin sections through the Harding Sandstone and it is assumed that the extensive endoskeletal cartilages in PF 1795 are also formed from this tissue[5,56,58]. In our scan data these cartilages are clearly a different tissue to the dermal squamation, being solid and radiodense, without differentiated outer and inner layers, and penetrated by a dense, branching network of canals (Fig. 2d, S5). Higher resolution scans of PF 1795a show that this tissue's density varies, appearing slightly spongiose further away from the surface and the canal lumina (Fig. S3). A layer of this tissue close to the surface of the elements and around the canal lumina is slightly less radiodense and is riven by cracks. There is no evidence for chondrocyte spaces or spheritic growth, although these structures are likely below the resolution achieved in the data. However the tissue appears identical in the tomograms to what are likely isolated chunks of cartilage in the mash underlying the part of *Eriptychius* that is preserved in the matrix, and doesn't show any tissue structures associated with endochondral bone such as trabeculae. They are also certainly endoskeletal, based on their being formed from a distinct tissue to the dermal tesserae attached to their surface (e.g. Fig. 1e).

2. Phylogenetic analysis notes

The phylogenetic matrix used was modified from Miyashita *et al.*[45], itself modified from Miyashita *et al.*[46]. Modifications to taxa and characters are detailed below and were limited in scope to 'pteraspidomorph' taxa.

Taxa modifications:

Removed: Arandaspida, Heterostraci,

Added: *Anglaspis, Eriptychius, Errivaspis, Sacabambaspis*

Modified: *Astraspis, Athenaegis*

Taxon	Source
Anglaspis	Kiaer 1932[59], Blieck and Heintz 1983[60], Keating *et al.* 2015[61]
Astraspis	Walcott 1892[48], Elliott 1987[22], Sansom 1997[25]
Athenaegis	Soehn and Wilson 1990[56]
Eriptychius	Walcott 1892[54], Denison 1967[5], Smith and Hall 1990[57], FMNH PF 1795
Errivaspis	White 1937[63], Keating et al 2015[61]*
Sacabambaspis	Gagnier et al 1986[29], Gagnier 1993a[31],b[32], Sansom *et al.* 2005[64], Pradel et al 2007[34]

*Pteraspids in Keating used as proxy for *Errivaspis* histology

Character/coding modifications

Character 3. *Distinct prechordal head: 0, absent or weakly developed; 1, prominent.*

In previous versions of this matrix *Sacabambaspis* was scored 1 based on a heterostracan-like anatomical interpretation (see discussion in supplement of Miyashita *et al.* 2019[45]). However, as discussed in the main text, the incoherency of pteraspidomorphs and the dissimilarity of the placement of the orbits in *Sacabambaspis* compared to *Eriptychius*, *Astraspis*, and heterostracans leaves open the possibility that the orbital position in *Sacabambaspis* reflects a weakly developed prechordal head rather than being derived from a heterostracan-like form. Thus we score *Sacabambaspis* 0 which we believe to be more consistent with the observed state in the fossils. We note though that either of these interpretations are plausible until more is known about the internal anatomy of arandaspids.

Character 4. *Morphologically distinct cerebellum with corpus cerebelli: 0, absent; 1, present*. Following Miyashita *et al.* [45,46] scoring for heterostracans *Anglaspis* has been scored 1, but note it has been disputed whether this constitutes the cerebellum (summarised in Janvier 1996[2] pg. 273).

Character 12. *Pineal organ (extra-ocular photoreceptor region expressing pineal opsins): 0, absent; 1, present.*

We scored *Eriptychius* as present based on the pineal opening tentatively identified in the median dorsal cartilage. For *Sacabambaspis* we have followed the interpretation of Gagnier [31], however see Zhang *et al.* [53] for an alternative anatomical interpretation

Character 13. *Pineal opening: 0, covered; 1, uncovered*

We scored *Eriptychius* as unknown as it is unclear whether the tessera overlying the median dorsal plates is in life position or whether it has an opening in.

Character 19. *Nasohypophyseal opening: 0, single; 1, paired*

Miyashita *et al.*[45,46] coded this character on the basis of the number of olfactory openings. Based on Gagnier's[31] interpretation *Sacabambaspis* had two openings; we have coded it as paired. Heterostracan taxa are coded ?: the anatomy nasohypophyseal openings has never been conclusively shown.

Character 26. *Eyes with pigmented retinal epithelium: 0, absent; 1, present*

Eriptychius is assumed to have pigmented eyes on the basis of the orbits and orbital scales and so we scored it present.

Character 27. *Eyes: 0, exposed; 1, covered by dermis; 2, covered by trunk muscles*

This is unknown in all fossil vertebrates. For consistency's sake with the other fossil vertebrates in the analysis, and as a full reworking of the phylogenetic dataset is beyond the scope of this paper, here *Eriptychius, Sacabambaspis, Astraspis* and heterostracan taxa are assumed to have uncovered eyes and we have scored them absent in keeping with the scoring approach of Miyashita *et al.* [45,46].

Character 32. *Eyes: 0, laterally placed (interorbital distance equal to width of head at that position); 1, close together near midline (interorbital distance substantially less than width of head at that position); 2, on prominent eyestalk.*

Although the eyes are oriented anteriorly rather than laterally *Sacabambaspis* has been scored 0 as the eyes are placed laterally relative to the head width at that position. We have scored *Eriptychius* absent based on the inferred position of the orbits and orbital scales.

Character 33. *Cartilaginous otic capsules: 0, absent; 1, present.*

Cartilaginous otic capsules are not preserved in *Eriptychius* PF 1795. However, it is unclear whether they were present in life as they could have either been unmineralized and thus not fossilised, or alternatively lost prior due to disruption to the carcass during fossilisation. Thus we have scored this character as unknown. We have also changed the scoring for heterostracan taxa from 1 to ?

Character 38. *Endolymphatic duct: 0, is blind; 1, opens externally.*

Based on articulated headshields that have no evidence of an endolymphatic opening we have scored both *Sacabambaspis* and *Astraspis* as blind (0) for this character. In *Eriptychius* this is unknown. As with the pineal for *Sacabambaspis* we have followed the interpretation of Gagnier[31], but see Zhang *et al.* [53] for an alternative anatomical interpretation

Character 52: *Single confluent branchial opening, absent = 0, present = 1*

We have changed the scoring for *Athenaegis* from 0 to ? for this and contingent characters as the condition is described as unknown by Soehn and Wilson [56].

Character 54. *Branchial openings: 0, spaced accordingly with dimensions of branchial cavities; 1, packed closely together; 2, organized into multiple parallel rows*

This was scored inapplicable for heterostracans with a single confluent branchial opening, following the approach for hagfishes with a single branchial opening.

Character 63. *Position of mouth: 0, terminal; 1, subterminal.*

The mouth in *Athenaegis* is subterminal , although it lacks the elongate rostrum of pteraspid heterostracans the oral plates are positioned ventrally relative to the rostral plate Soehn and Wilson [56]. Scoring is changed from 0 to 1.

Character 64. *Epidermal oral cirri: 0, absent; 1, present.*

This is unknowable in most fossil vertebrates. For consistency's sake with the other fossil vertebrates in the analysis, and as a full reworking of the phylogenetic dataset is beyond the scope of this paper we have scored *Eriptychius, Sacabambaspis, Astraspis,* and heterostracan taxa absent in keeping with the scoring approach of Miyashita *et al.* [45,46].

Character 65. *Postoptically derived ectomsenchyme anterior to mandibular arch gives rise to palatal structures that: 0, meet at midline under nasal/nasohypophyseal organs; 1, meet at dorsal midline anterior to nasohypophyseal organs and form a prominent oral roof.*

We consider the palate of *Sacabambaspis* and heterostracans to be insufficiently well-characterised to score this character and so have scored them as unknown.

Character 82. *Distinct dorsal fin: 0, absent; 1, present.*

This is judged to be absent in *Astraspis*, in which two specimens show the articulated dorsal headshield with no evidence for a dorsal fin[30,33]; we have scored it absent.

Character 91. *Tail shape: 0, no distinct lobes developed; 1, ventral lobe much larger than dorsal; 2, dorsal lobe much larger than ventral; 3, dorsal and ventral lobes almost equally developed*

We have scored this 1 for *Anglaspis* and *Errivaspis*

Character 92 Chordal disposition relative to tail development: 0, isochordal; 1, hypochordal; 2, hyperchordal

We scored this 1 for *Anglaspis* and *Errivaspis* but ? for *Athenaegis*

Character 95. *Cellular bone: 0, absent; 1, present*

We have scored *Athenaegis* ? for this and subsequent characters , as no histological detail is known for the taxon.

Character 98. *Calcified cartilage: 0, absent; 1, present.*

We have scored this present in *Eriptychius*, following Denison[8], as we consider it the most plausible interpretation, but we note that the identity of the endoskeletal tissue is not firmly established. *Sacabambaspis* and *Astraspis* are scored ? as only dermal elements are preserved in fossils: the taphonomic loss of calcified cartilage cannot be ruled out.

Character 109. *Calcification/ossification occurs in endoskeleton: 0, absent; 1, present.*

Following the same approach as for character 98 we have scored *Sacabambaspis* and *Astraspis* unknown for this character and *Eriptychius* present.

Character 110. *Calcification/ossification occurs in exoskeleton: 0, absent; 1, present.*

We have scored *Sacabambaspis* present for this character.

Character 118. *Oak-leaf-shaped tubercles: 0, absent; 1, present.*

Although in previous versions of this matrix they were scored present, oak-leaf shaped tubercles are absent in *Astraspis* [30,33]. They are also absent in *Eriptychius*[8]. We have scored both taxa absent for this character. At present the only taxon with this character present is *Sacabambaspis,* so the character is uninformative but is kept in to preserve the character number scheme.

Character 127. *Dermal head covering in adult state: 0, micromeric; 1, large (macromeric) dermal plates or shield.*

In previous versions of this matrix (Miyashita *et al.*[45,46]) *Astraspis* is scored as micromeric for this character despite the fact that is has a dermal skeleton formed from polyodontode tesserae[30,33]. For the sake of consistency with other scorings we have kept this and scored *Astraspis* and *Eriptychius* as micromeric, but we note that mesomery is arguably as similar to macromery as it is to micromery.

Character 135. *Denticulate/cuspidate elements of feeding apparatus, perioral: 0, absent; 1, present*

Following the original scoring strategy for this character we have scored heterostracans as 1 based on the ornamentation of their oral plates, and *Eriptychius* 1 due to denticles on the surface of the plates adjacent to the mouth.

Character 140. *Feeding apparatus forming a pulley-like system of cartilages and protractor-retractor complex in mandibular arch: 0, absent; 1, present*

This is unknown in heterostracans, where no cartilages are preserved, and is changed from 0 to ?

Character 152. *Braincase with lateral walls: 0, absent; 1, present.*

Due to the preservation of only the preorbital region in *Eriptychius* we have coded it unknown for this character.

Character 156. *Barbels supported by cartilages: 0, absent; 1, present.*

Cartilages are not preserved in heterostracans, so this is scored ? for those taxa.

3. Supplementary references

56. Ørvig, T. Histologic studies of ostracoderms, placoderms and fossil elasmobranchs. 6. Hard tissues of Ordovician vertebrates. *Zoologica Scripta* **18**, 427–446 (1989).

57. Smith, M. M. & Hall, B. K. Vertebrate skeletogenic and odontogenic tissues. *Biological Reviews* **65**, 277–373 (1990).

58. Halstead, L. B. The Heterostracan Fishes. *Biological Reviews* **48**, 279–332 (1973).

59. Kiaer, J. The Downtonian and Devonian vertebrates of Spitsbergen IV Suborder Cyathaspida. *Skrifter om Svalbard og Ishavet* (1932).

60. Blieck, A. & Heintz, N. The cyathaspids of the Red Bay Group (Lower Devonian) of Spitsbergen. *Polar Research* **1**, 49–74 (1983).

61. Keating, J. N., Marquart, C. L. & Donoghue, P. C. J. Histology of the heterostracan dermal skeleton: Insight into the origin of the vertebrate mineralised skeleton. *Journal of Morphology* **276**, 657–680 (2015).

62. Soehn, K.L. and Wilson, M.V.H. A complete, articulated heterostracan from Wenlockian (Silurian) beds of the Delorme Group, Mackenzie Mountains, Northwest Territories, Canada. *Journal of Vertebrate Paleontology* **10** 405-419 (1990)

63. White, E.I. The ostracoderm Pteraspis KNER and the relationships of the agnathous vertebrates. *Phil. Trans. Roy. Soc. Lon. B* **225** 381-456 (1935)

64. Sansom, I.J., Donoghue, P.C.J., Albanesi, G. Histology and affinity of the earliest armoured vertebrates. *Biology Letters*. **1** 446-449 (2005)

4. Caption for Supplementary Video 1

Video showing our 3D models of the partially articulated *Eriptychius americanus* specimen PF 1795 we describe in the main text. The specimen is shown with all identified elements (from PF 1795 a + b) digitally placed relative to the rock matrix of PF 1795b, then the entire articulated specimen, then the specimen with dermal elements removed, and finally our reconstruction of the arrangement of cartilages.
