# Supplementary material for: The oldest three-dimensionally preserved vertebrate neurocranium
Source: Nature. 2023 Sep 20;621(7980):782–7. doi: 10.1038/s41586-023-06538-y (PMC10533405; doi:10.1038/s41586-023-06538-y)
Supplement: Supplementary file 2 — Reporting Summary [file 41586_2023_6538_MOESM2_ESM.pdf]

nature portfolio

Corresponding author(s): Richard P. Dearden

Last updated by author(s): Aug 1, 2023

Reporting Summary

Nature Portfolio wishes to improve the reproducibility of the work that we publish. This form provides structure for consistency and transparency in reporting. For further information on Nature Portfolio policies, see our Editorial Policies and the Editorial Policy Checklist.

Statistics

For all statistical analyses, confirm that the following items are present in the figure legend, table legend, main text, or Methods section.

n/a	Confirmed	
☒	☐	The exact sample size (n) for each experimental group/condition, given as a discrete number and unit of measurement
☒	☐	A statement on whether measurements were taken from distinct samples or whether the same sample was measured repeatedly
☒	☐	The statistical test(s) used AND whether they are one- or two-sided<br>*Only common tests should be described solely by name; describe more complex techniques in the Methods section.*
☒	☐	A description of all covariates tested
☒	☐	A description of any assumptions or corrections, such as tests of normality and adjustment for multiple comparisons
☒	☐	A full description of the statistical parameters including central tendency (e.g. means) or other basic estimates (e.g. regression coefficient) AND variation (e.g. standard deviation) or associated estimates of uncertainty (e.g. confidence intervals)
☒	☐	For null hypothesis testing, the test statistic (e.g. F, t, r) with confidence intervals, effect sizes, degrees of freedom and P value noted<br>*Give P values as exact values whenever suitable.*
☐	☒	For Bayesian analysis, information on the choice of priors and Markov chain Monte Carlo settings
☒	☐	For hierarchical and complex designs, identification of the appropriate level for tests and full reporting of outcomes
☒	☐	Estimates of effect sizes (e.g. Cohen's d, Pearson's r), indicating how they were calculated

Our web collection on statistics for biologists contains articles on many of the points above.

Software and code

Policy information about availability of computer code

Data collection	not applicable
Data analysis	For analysis of the image data we used the paid software Mimics v.25 (Materialise) to make 3D models and the free software Blender (blender.org) to acquire images. For the phylogenetic analysis we used the software tnt and MrBayes.

For manuscripts utilizing custom algorithms or software that are central to the research but not yet described in published literature, software must be made available to editors and reviewers. We strongly encourage code deposition in a community repository (e.g. GitHub). See the Nature Portfolio guidelines for submitting code & software for further information.

Data

Policy information about availability of data

All manuscripts must include a data availability statement. This statement should provide the following information, where applicable:

- Accession codes, unique identifiers, or web links for publicly available datasets
- A description of any restrictions on data availability
- For clinical datasets or third party data, please ensure that the statement adheres to our policy

The CT scan data this work is based on is available via Morphosource via the following links: PF 1795a (https://doi.org/10.17602/M2/M510644), PF 1795b (https://doi.org/10.17602/M2/M510665). A mesh incorporating all 3D surface models generated in this study is made available via Morphosource at the following link

(https://doi.org/10.17602/M2/M542071). Individual 3D meshes and the phylogenetic matrix are made available on figshare at the following links (https://doi.org/10.6084/m9.figshare.23726487).

Research involving human participants, their data, or biological material

Policy information about studies with human participants or human data. See also policy information about sex, gender (identity/presentation), and sexual orientation and race, ethnicity and racism.

Reporting on sex and gender	not applicable
Reporting on race, ethnicity, or other socially relevant groupings	not applicable
Population characteristics	not applicable
Recruitment	not applicable
Ethics oversight	not applicable

Note that full information on the approval of the study protocol must also be provided in the manuscript.

Field-specific reporting

Please select the one below that is the best fit for your research. If you are not sure, read the appropriate sections before making your selection.

☐ Life sciences ☐ Behavioural & social sciences ☒ Ecological, evolutionary & environmental sciences

For a reference copy of the document with all sections, see nature.com/documents/nr-reporting-summary-flat.pdf

Ecological, evolutionary & environmental sciences study design

All studies must disclose on these points even when the disclosure is negative.

Study description	We used computed tomography to image a fossil fish and then segmented the data to create 3D models of its skeleton. We used these as the basis for interpretation and phylogenetic analysis.
Research sample	Eriptychius americanus specimen from the Field Museum, Chicago PF 1795
Sampling strategy	Only one specimen exists.
Data collection	The specimen was CT scanned at the UChicago PaleoCT facility by April Neander, and subsequently at Bristol University, UK by Sam Giles and Liz Martin-Silverstone
Timing and spatial scale	This study was carried out between June 2022 and March 2023
Data exclusions	No data were excluded
Reproducibility	CT scan settings are given in the materials and methods, the scan data and phylogenetic data is provided freely allowing others to reproduce the study.
Randomization	This is not relevant as only one specimen was used
Blinding	Blinding is irrelevant as no blinding was possible

Did the study involve field work? ☐ Yes ☒ No

Reporting for specific materials, systems and methods

We require information from authors about some types of materials, experimental systems and methods used in many studies. Here, indicate whether each material, system or method listed is relevant to your study. If you are not sure if a list item applies to your research, read the appropriate section before selecting a response.

Materials & experimental systems

n/a	Involved in the study
☒	☐ Antibodies
☒	☐ Eukaryotic cell lines
☐	☒ Palaeontology and archaeology
☒	☐ Animals and other organisms
☒	☐ Clinical data
☒	☐ Dual use research of concern
☒	☐ Plants

Methods

n/a	Involved in the study
☒	☐ ChIP-seq
☒	☐ Flow cytometry
☒	☐ MRI-based neuroimaging

Palaeontology and Archaeology

Specimen provenance	The specimen is held in the Field Museum, Chicago, USA and was taken out on loan for the duration of the study.
Specimen deposition	The specimen continues to be held at the Field Museum
Dating methods	No new dates are provided

☐ Tick this box to confirm that the raw and calibrated dates are available in the paper or in Supplementary Information.

Ethics oversight	None required, material already held in collections

Note that full information on the approval of the study protocol must also be provided in the manuscript.
